# Resilience: a nationwide study of medical educators

**DOI:** 10.15694/mep.2019.000020.1

**Published:** 2019-01-29

**Authors:** Linda Chan, Ashley Dennis

**Affiliations:** 1 In His Image Family Medicine Residency; 2 Billings Clinic

**Keywords:** Resilience, medical educators, learners, depression, contextual, sociocultural.

## Abstract

This article was migrated. The article was marked as recommended.

**Background:** Medical educators have the potential to play an important role in addressing resilience issues across the medical education continuum. Yet, limited research has explored this cohort, particularly in the UK context. This study aims to address this gap by exploring resilience and the factors that relate to resilience in UK medical educators.

**Methods:** A cross-sectional online questionnaire was employed. It contained the validated Connor-Davidson Resilience Scale 25 (CD-RISC-25) and Patient Health Questionnaire-8 (PHQ-8), for determining medical educator resilience and depression levels respectively. Sociodemographic characteristics were also collected to identify potential factors associated with resilience. Finally, participants identified factors that facilitated or undermined their resilience through open-ended questions. Data was analysed using quantitative (e.g. multiple regression analysis), and qualitative (i.e. thematic framework analysis) approaches.

**Results:** Among 244 UK participants, the mean CD-RISC-25 score was 72.9 and standard deviation 10.3. The PHQ-8 score was the only significant negative predictor identified (B = -1.22, p < 0.001). Other sociodemographic variables examined were not found to be predictors of CD-RISC-25 scores. Educators identified multidimensional factors influencing their resilience, but predominately viewed internal factors as being supportive, and external factors as undermining.

**Conclusions:** Medical educators in this study had resilience levels that were comparable to other population samples. The study also highlights the importance of considering external factors, such as contextual and sociocultural influences, in addition to individual factors when addressing resilience.

## Introduction

Among medical learners and practitioners, alarming rates of psychological distress have garnered attention in recent years ( Dyrbye, Thomas and Shanafelt, 2006; Shanafelt *et al.*, 2011; Dyrbye *et al.*, 2014; Hope and Henderson, 2014; Mata *et al.*, 2015; Rotenstein *et al.*, 2016). Further concern has arisen as literature highlights the potential negative implications of distress in physicians and trainees on patient care, as well as the healthcare system ( Shanafelt *et al.*, 2002; West *et al.*, 2006; Shanafelt *et al.*, 2010; Panagioti *et al.*, 2017; West, Dyrbye and Shanafelt, 2018). In response, resilience has emerged as a prominent concept in the arena of clinician and learner well-being ( Howe, Smajdor and Stockl, 2012; Southwick and Charney, 2012).

Resilience has multiple definitions within existing literature ( Luthar, Cicchetti and Becker, 2000; Aburn, Gott and Hoare, 2016; Rogers, 2016).This study incorporates two overlapping views of resilience to provide theoretical support. First, Windle’s (2011) systematic review defines resilience as, ‘the process of effectively negotiating, adapting to, or managing significant sources of stress or trauma’ ( Windle, 2011, pp. 163). This definition highlights the dynamic and complex nature of resilience, which depends not only on the individual, but also on resources available through his or her community, society, and culture ( Windle, 2011). Linked to this holistic approach is the biopsychosocial-spiritual framework ( Sulmasy, 2002), which considers the biological, psychological, social, and spiritual facets of resilience.

### Resilience in medical education

Globally, research investigating resilience in medical learners has proliferated ( Haglund *et al.*, 2009; Peng *et al.*, 2012; Cooke, Doust and Steele, 2013; Rahimi *et al.*, 2014; Olson, Kemper and Mahan, 2015; Tempski *et al.*, 2015; Bacchi and Licinio, 2017; Dyrbye, Shanafelt *et al.*, 2017; Eley *et al.*, 2017; Houpy *et al.*, 2017; Oliveira, Machado and Aranha, 2017). In contrast, limited empirical studies have been conducted among medical educators ( Cora-Bramble, Zhang and Castillo-Page, 2010; Sood *et al.*, 2011; DeCastro *et al.*, 2013; Sood *et al.*, 2014; Porter *et al.*, 2018). Compared to a wider group of clinicians, their educational focus uniquely positions them to cultivate resilience in their learners, as well as themselves. For example, this may be achieved through instruction in the formal curriculum, role-modelling in the informal, and creating a supportive learning culture as part of the hidden curriculum ( Cruess, Cruess and Steinert, 2008; Goodyear, 2014; General Medical Council, 2015a; Abaza and Nelson, 2018). Consequently, examining the resilience of medical educators as a specific cohort is important.

In the broader medical education literature, a number of studies have investigated factors relating to resilience. Several have identified negative associations between resilience and facets of distress such as depression ( Shi *et al.*, 2016), burnout ( Cooke, Doust and Steele, 2013), stress ( Rahimi *et al.*, 2014), and general mental health problems ( Peng *et al.*, 2012). However, we are not aware of any studies exploring facets of distress, such as depression, in relation to resilience in medical educators specifically. When considering the relationship between resilience and sociodemographic variables, inconsistencies have been reported ( Davidson and Connor, 2015). For example, research involving Chinese ( Peng *et al.*, 2012), Canadian ( Rahimi *et al.*, 2014), and U.S. ( Houpy *et al.*, 2017) medical students found male students as having statistically higher resilience scores than females. Whereas, Elizondo-Omaña *et al.* (2010) did not observe such differences in resilience scores by gender in Mexican medical students. For medical educators, Porter and colleagues (2018) found no associations between the resilience level of U.S. family medicine programme directors and their demographic background, including gender.

Psychological and demographic factors aside, health professionals have also reported a wide range of internal and external factors that facilitate and hinder their resilience. Examples include work-intensification, challenging patients, preparation for practice, work-life balance, support from family and friends, connection with colleagues, personal characteristics (e.g. self-reflection, help seeking), and spirituality ( McCann *et al.*, 2013; Zwack and Schweitzer, 2013; Goodyear, 2014; Matheson *et al.*, 2016; Robertson *et al.*, 2016; Cheshire *et al.*, 2017). However, there is a dearth of research exploring factors that medical educators identify as being related to their resilience.

This study aims to extend the medical education literature by addressing several gaps. First, of the limited research examining medical educator resilience, none was found in the UK context and none explored resilience levels of this group in a way that would enable comparisons with other populations. Second, there are few studies exploring psychological and sociodemographic factors relating to resilience in medical educators. Finally, there is limited research exploring factors that medical educators identify as influencing their resilience across the biopsychosocial-spiritual framework ( Sulmasy, 2002).

In order to address these gaps, this study first establishes baseline resilience scores of UK medical educators. It then investigates the relationships between educators’ resilience levels, their sociodemographic characteristics, and depression scores. Finally, the study examines what factors medical educators regard as being beneficial or detrimental to their own resilience. Our research questions are as follows:

RQ1: How resilient are UK medical educators, and what factors are associated with their resilience?

RQ2: What factors do medical educators perceive as promoting or undermining their resilience?

## Methods

### Study and questionnaire design

We adopted a pragmatic paradigm ( Creswell, 2014), and designed a concurrent embedded mixed methods study to address our research questions ( Creswell and Plano Clark, 2011). Here, a qualitative strand gathering educators’ views through open-ended questions, was included within a larger quantitative, cross-sectional questionnaire ( Creswell and Plano Clark, 2011). The University of Dundee’s Research Ethics Committee approved this study. An anonymous, self-administered online questionnaire was employed due to its efficiency and cost-effectiveness for gathering data from eligible educators located across the UK ( Boynton and Greenhalgh, 2004; Artino *et al.*, 2014). It was developed using BOS ( https://www.onlinesurveys.ac.uk), and incorporated two validated instruments, in addition to open-ended and sociodemographic questions.

The validated 25-item Connor-Davidson Resilience Scale (CD-RISC-25) was included for self-assessing resilience ( Connor and Davidson, 2003). Total scores range from 0 to 100, with higher scores reflecting greater resilience ( Davidson and Connor, 2015). A methodological review of 15 resilience measures identified the CD-RISC-25 as one of three instruments with the best psychometric ratings overall ( Windle, Bennett and Noyes, 2011). Internal consistency in terms of Cronbach’s alpha was 0.89, and test-retest reliability 0.87 in the original validation study ( Connor and Davidson, 2003). Its convergent, divergent, and construct validity have also been established ( Connor and Davidson, 2003; Davidson and Connor, 2015).

The Patient Health Questionnaire-8 (PHQ-8) was used for self-reporting depressive symptoms, with scores ranging from 0 to 24 ( Kroenke *et al.*, 2009). It incorporates eight items from the PHQ-9, and is suited for self-administered questionnaires when intervention for positive responses to item nine on suicidal ideation or self-harm is precluded ( Kroenke and Spitzer, 2002). Prior research has established the PHQ-8’s construct validity ( Pressler *et al.*, 2011), and internal consistency ( Pressler *et al.*, 2011; Ritter *et al.*, 2014).

Sociodemographic details were collected to characterise our sample, as well as to explore whether any relationships existed between them and resilience. These included: (i) age, (ii) gender, (iii) race, (iv) relationship status, (v) years worked as a medical educator, (vi) professional background, and (vii) average hours worked per week. Furthermore, open-ended questions about educators’ views on resilience were incorporated (e.g. what factors enhance or undermine your resilience). Preliminary drafts of the online questionnaire were reviewed and feedback obtained from six medical educators in the development process.

### Procedures and participants

Power analysis indicated a minimum required sample size of 109 ( Cook and Hatala, 2015). Convenience sampling was used due to its cost-effectiveness, ease of implementation, and efficiency in achieving this sample size considering our resource constraints ( Tavakol and Sandars, 2014). Initially, all UK-based medical educators enrolled on the University of Dundee’s Centre for Medical Education postgraduate programs were recruited as they were reflective of the range of educators across the medical education spectrum and across multidisciplinary backgrounds. Educators based outside of the UK, or exclusively teaching healthcare professionals other than doctors were excluded. Participants were recruited using multiple strategies that contained links to the online questionnaire: (i) e-mails, (ii) social media, (iii) e-notices, and (iv) printed notices. A participant information sheet was either distributed with the e-mails, or was accessible from the questionnaire’s front page ( Sue and Ritter, 2007). Informed consent for voluntary participation was obtained electronically ( World Medical Association, 2013), and no financial rewards were offered for involvement. After three months, recruitment was widened to support meeting the sample size requirement. Electronic invitations were snowballed through the Academy of Medical Educators, Association for the Study of Medical Education, National Association of Clinical Tutors UK, Scottish Medical Education Research Consortium, and University of Dundee’s School of Medicine. The data collection time frame was April to September 2016.

### Quantitative analysis

Descriptive statistics were used to characterise our sample. Internal consistency of the CD-RISC-25 and PHQ-8 were evaluated using Cronbach’s alpha. The mean and standard deviation for the sample’s CD-RISC-25 scores were calculated as a measure of respondents’ resilience ( Davidson and Connor, 2015). To determine which factors were associated with resilience, a multiple regression analysis was conducted using the general linear model procedure ( Taylor, 2011). This examined the relationship between CD-RISC-25 score as the dependent variable, and eight independent variables (seven sociodemographic factors plus the PHQ-8 score) which were entered into the model simultaneously. This process mitigated the influence of confounding and effect modification on relationships between variables ( Thompson, 2011). If certain categorical variables had less than five subjects per group, this was either combined with the ‘Other’ group if available, or collapsed together to form a new ‘Other’ group. This ensured a minimum of two subjects per variable in the model was exceeded, to allow accurate estimation of regression coefficients ( Austin and Steyerberg, 2015). Given the number of comparisons conducted, a Bonferroni adjustment was also made to control the overall type I error rate (α=0.004) ( Mundfrom *et al.*, 2006). All statistical tests were performed using IBM SPSS Statistics for Windows Version 22.0 (IBM Corp., Armonk, NY, USA).

### Qualitative analysis

Textual data gathered from open-ended questions were anonymised, and analysed using thematic framework analysis ( Ritchie and Spencer, 1994). First, this involved familiarisation. Second, a representative data sample was used to identify major themes and subthemes. We then compared, contrasted, and negotiated identification of an initial thematic framework (LC, AD). Inductive data analysis and a deductive approach based on the biopsychosocial-spiritual model ( Sulmasy, 2002) were used over five iterations before this framework was finalised. Third, the framework was employed by LC to code all the qualitative data using ATLAS.ti for Mac Version 7.0 (Scientific Software Development GmbH, Berlin, Germany). During this indexing process, any discrepancies noted between the data and framework were resolved between the authors. Fourth, the data was ‘charted’ or reorganised according to the themes and subthemes in the framework using ATLAS.ti. Finally, data patterns were mapped, synthesised, and interpreted.

## Results/Analysis

### Characteristics of respondents and instrument scores

Overall, 247 educators participated, but three did not meet inclusion criteria. The final number of individuals who received the invitation was unknown because of the recruitment techniques, and thus, a response rate could not be calculated. Internal consistency (Cronbach’s alpha) for the CD-RISC-25 (0.88) and PHQ-8 (0.83) indicated adequate reliability ( Lance, Butts and Michels, 2006). These values were also comparable to those reported in the literature ( Connor and Davidson, 2003; Pressler *et al.*, 2011). Sociodemographics and instrument scores for participants included in the analysis are summarised in table 1.

**Table 1.** Characteristics of respondents, their CD-RISC-25 and PHQ-8 scores

Respondent characteristics (N = 244)		
Geographic location ^*^, no. (%)		
	England	150 (61.5)
	Northern Ireland	4 (1.6)
	Scotland	69 (28.3)
	Wales	8 (3.3)
**Gender, no. (%)**		
	Female	142 (58.2)
	Male	100 (41.0)
	Prefer not to say	2 (0.8)
**Age in years, no. (%)**		
	20 - 29	21 (8.6)
	30 - 39	49 (20.1)
	40 - 49	69 (28.3)
	50 - 59	74 (30.3)
	≥ 60	30 (12.3)
	Prefer not to say	1 (0.4)
Race ^*^, no. (%)		
	Afro-Caribbean	1 (0.4)
	Asian	20 (8.2)
	Caucasian	202 (82.8)
	Mixed/multiple	4 (1.6)
	Other	8 (3.3)
	Prefer not to say	8 (3.3)
Relationship status ^*^, no. (%)		
	Single	16 (6.6)
	Partnered	45 (18.4)
	Married	173 (70.9)
	Separated	2 (0.8)
	Divorced	3 (1.2)
	Widowed	1 (0.4)
	Prefer not to say	3 (1.2)
Professional background ^*^, no. (%)		
	Allied health	8 (3.3)
	Dentistry	4 (1.6)
	Medicine	206 (84.4)
	Nursing	8 (3.3)
	Veterinary medicine	1 (0.4)
	Other	16 (6.6)
**Years of experience as medical educator, no. (%)**		
	0 - 4	53 (21.7)
	5 - 9	49 (20.1)
	10 - 14	48 (19.7)
	15 - 19	30 (12.3)
	≥ 20	64 (26.2)
Average hours worked per week ^*^, no. (%)		
	≤ 19	13 (5.3)
	20 - 39	42 (17.2)
	40 - 59	149 (61.1)
	60 - 79	28 (11.5)
	≥ 80	9 (3.7)
**CD-RISC-25 score ( *n* = 232) ^†^, mean (SD)** 72.9 (10.3)		
**PHQ-8 score ( *n* = 236) ^†^, mean (SD)** 3.1 (3.5)		

CD-RISC-25 = 25-item Connor-Davidson Resilience Scale; PHQ-8 = Patient Health Questionnaire-8; SD = standard deviation.

^*^ Numbers may not add up to 244 and percentages may not add up to 100% due to missing data.

^†^ *n* is less than 244 due to missing data.

**RQ1: How resilient are UK medical educators, and what factors are associated with their resilience?**

Participants’ CD-RISC-25 scores ranged from 42 to 98, with a mean (SD) of 72.9 (10.3) as per table 1. Their mean PHQ-8 score was 3.1, with a standard deviation of 3.5. The multiple regression analysis demonstrated that the CD-RISC-25 resilience score was significantly predicted by only the PHQ-8 depression score (B = -1.22, p < 0.001), with the model explaining 23.6% of the variance ( *R* ^2^ = 0.236, *F*(13,195) = 4.64, p < 0.001). The seven sociodemographic variables were not found to be significant predictors of CD-RISC-25 score. Findings from the multiple regression analysis are summarised in table 2.

**Table 2.** Summary of the multiple regression analysis for variables predicting CD-RISC-25 score

Variable	B (95% CI)	SE B	*t*	p-value
**Constant**	72.32	4.11	17.58	< 0.001
	(64.21, 80.43)			
**PHQ-8 score**	-1.22	0.19	-6.58	< 0.001
	(-1.59, -0.86)			
**Age**	0.29	0.95	0.30	0.762
	(-1.59, 2.17)			
**Average hours worked per week**	0.50	0.89	0.56	0.576
	(-1.26, 2.27)			
**Years of experience as medical educator**	0.59	0.70	0.83	0.405
	(-0.80, 1.98)			
**Gender (ref=Male)**				
Female	1.79	1.39	1.28	0.201
	(-0.96, 4.53)			
**Professional background (ref=Medicine)**				
Allied health	2.30	3.56	0.64	0.520
	(-4.73, 9.32)			
Nursing	-1.41	3.62	-0.39	0.698
	(-8.55, 5.73)			
Other	0.85	2.44	0.35	0.728
	(-3.97, 5.67)			
**Race (ref=Caucasian)**				
Asian	0.21	2.45	0.09	0.932
	(-4.62, 5.04)			
Other	5.74	2.98	1.93	0.055
	(-0.14, 11.61)			
**Relationship status (ref=Married)**				
Single	-1.61	2.72	-0.59	0.554
	(-6.98, 3.75)			
Partnered	-1.22	1.76	-0.70	0.487
	(-4.69, 2.24)			
Other	3.78	4.21	0.90	0.370
	(-4.52, 12.09)			

CI = confidence interval; SE = standard error; ref = reference.

*R* ^2^ = 0.236 (Adjusted *R* ^2^ = 0.185).

**RQ2: What factors do medical educators perceive as promoting or undermining their resilience?**

Within the qualitative data, educators mentioned two overarching themes when reflecting on factors that influenced their resilience. First, they identified internal aspects relating to the individual. This encompassed biological (e.g. health), psychological (e.g. emotional regulation), and spiritual (e.g. religion) facets:

*Active, healthy lifestyle. Positive personal qualities relating to a sense of humour and positive outlook. (P #222)*

Second, they highlighted external aspects relating to the socio-environmental context, including interpersonal, organisational, and societal elements:

*Intimidating behaviour. Non-constructive negative feedback. Refusal to provide advice or guidance from supervisors when asked for. (P#61)*

Furthermore, promoting and undermining factors largely mirrored each other. For instance, one participant highlighted her social network as building her resilience:

*Supportive family & friends, especially a healthy mixture of medical and non-medical friends. (P#166)*

However, strain within the social network could also undermine resilience:

*..when parents get ill and take the emotional energy that I would normally use to cope with work but have to cope with looking after them instead. When my child is ill and I have to take time off work when I can see there is no spare time in my week and my colleagues have no capacity to help either. (P#92)*

A key pattern that arose across the data was that resilience promoting factors were predominantly reported as being internal, for example, self-regulation of emotion as depicted by one educator:

*..being present emotionally and cognitively to whatever is happening whilst being able to distance myself sufficiently from emotions to be able to regulate or play (in an improvisational sense) with them. This is what I call being empathetically mindful. (P#194)*

Whereas, external aspects were mainly raised as being deleterious:

*The entire system in which we work is a culture of one-up-manship and blame - particularly blaming individual junior doctors for the failure - the only feedback we receive much of the time is that we are not working fast enough. (P#198)*

In addition to characterising the adversity that drained their resilience, respondents also identified that the timing and concurrence of multiple challenges were undermining:

*Too much adversity in one go can overwhelm a person and leave them feeling unable to cope which undermines resilience instead of building it up. (P#208)*

## Discussion

To our knowledge, this is the first empirical study exploring resilience in a UK-wide cohort of medical educators. Using the CD-RISC-25, participants’ resilience levels appeared to be comparable to those found in other studies conducted in the UK involving forensic and legal medicine professionals ( Horvath and Massey, 2018), military personnel ( Matthew *et al.*, 2015), and adults in the general community ( Petros, Opacka-Juffry and Huber, 2013). The resilience levels of respondents also appeared to be in line with U.S. populations reported in the original CD-RISC-25 validation study ( Connor and Davidson, 2003). Furthermore, the average PHQ-8 score suggested that most participants were in the range representing no significant depressive symptoms (i.e. scores less than five; Kroenke *et al.*, 2009). These findings are interesting as unlike other studies demonstrating that medical learners and practitioners might be at risk populations with higher rates of depression and other indicators of psychological distress ( Dyrbye, Thomas and Shanafelt, 2006; Dyrbye *et al.*, 2014; Hope and Henderson, 2014; Mata *et al.*, 2015; Rotenstein *et al.*, 2016), this study suggests that medical educators may not necessarily be at higher risk.

Increasing depressive symptoms were associated with decreasing resilience levels in medical educators. This extends the literature which has found a negative relationship between resilience and depression symptoms in other populations including healthcare professionals ( Rossouw *et al.*, 2013; Hegney *et al.*, 2015; Shi *et al.*, 2016). Further, these findings add to the evidence base of negative associations determined between resilience, and other facets of psychological distress such as burnout ( Cooke, Doust and Steele, 2013), stress ( Rahimi *et al.*, 2014), as well as mental health problems ( Peng *et al.*, 2012) reported in the wider medical education literature.

The seven sociodemographic variables examined were not found to be significant predictors of the CD-RISC-25 score. This aligns with other work in Mexican medical students ( Elizondo-Omaña *et al.*, 2010), South African physicians ( Rossouw *et al.*, 2013), and in the broader U.S. population ( Connor and Davidson, 2003), where no differences in mean CD-RISC-25 score were identified by the sociodemographic factors studied. Although they used the Brief Resilience Scale rather than the CD-RISC-25, Porter *et al*. (2018) also did not find associations between the resilience level and demographics of U.S. family medicine programme directors. However, other literature has found associations between CD-RISC-25 scores and various sociodemographic variables as summarised by Davidson and Connor (2015).

Participants identified multidimensional factors that influenced their resilience which aligns not only with Windle’s (2011) definition of resilience, but also with Sulmasy’s (2002) biopsychosocial-spiritual framework of resilience. Factors that were perceived as either promoting or undermining of resilience largely mirrored each other. Others have noted similar factors in UK healthcare professionals ( Platt *et al.*, 2015; Matheson *et al.*, 2016), and more specifically in medical educators from a U.S. context ( DeCastro *et al.*, 2013). Our findings are also consistent with recent arguments that supportive external factors can synergistically work with individual factors to foster resilience ( Balme, Gerada and Page, 2015; Matheson *et al.*, 2016; Cheshire *et al.*, 2017; Shanafelt and Noseworthy, 2017). Importantly, respondents mainly considered internal resources as being resilience promoting, while external factors were undermining. This pattern has not previously been described by medical educators, or to our knowledge, within the medical education literature more generally. This finding supports Hobfoll’s (2010) caution to not romanticise individual resilience, but balance this with addressing undermining issues within the external domains when developing interventions ( Balme, Gerada and Page, 2015; General Medical Council, 2015b; Dyrbye, Trockel *et al.*, 2017; Shanafelt and Noseworthy, 2017).

### Limitations

There are several limitations to this study that must be considered. First, convenience sampling may have resulted in self-selection and sampling bias ( Cohen, Manion and Morrison, 2011). The snowballing recruitment may have also compounded this tendency for self-selection. Therefore, it is important to cautiously consider the generalisability of the results across all UK medical educators. Additionally, the snowballing recruitment impeded our ability to calculate a response rate, and prevented data from non-responders from being considered. Nevertheless, our sample size exceeded the minimum determined by power analysis ( Cook and Hatala, 2015), and we had representation from educators based in all four UK countries across multidisciplinary backgrounds. Second, the CD-RISC-25 and PHQ-8 are both retrospective, self-reporting instruments that are susceptible to recall bias. This might lead to respondents under- or over-estimating their resilience levels and depressive symptoms. Further, the PHQ-8 in its resemblance to the PHQ-9, may have been recognised as a depression screening instrument, thereby potentially influencing participants’ answers. Third, our research represents a cross-sectional snapshot of a dynamic phenomenon, meaning causal conclusions cannot be drawn. For example, an individual’s resilience may fluctuate across his or her lifetime. Finally, as with all open-ended items in questionnaire-based research, we must acknowledge the limitations for clarifying underlying meanings or reasonings behind a response.

### Future directions for research

As highlighted earlier, this study provides a snapshot of medical educator resilience and depressive symptoms in the UK. First, it would be important to replicate this study on a larger scale, using random sampling techniques, and involving medical educator groups in other contexts globally to see if there are similar trends. Second, future prospective longitudinal trials investigating changes in resilience and depression symptoms over time could help determine whether causal relationships exist between these two constructs. They could also explore whether nurturing resilience may mitigate against the development of depression or other mental health conditions in this population. Furthermore, it would be important to directly compare medical educators to healthcare professionals and learners on these measures to examine whether there is something unique about medical educators as a group. Perhaps, for instance, there are aspects of the process of becoming and/or being a medical educator that foster resilience.

Our study also draws attention to the importance of considering both external and internal factors in developing resilience. Therefore, we suggest that further research should prioritise exploring external factors, and how these play a synergistic role with internal factors.

The qualitative data gathered in this study has offered a valuable starting point for understanding the multidimensional factors educators perceive as influencing their resilience. Future research could employ semi-structured interviews to explore in greater depth how the complex interactions between internal and external factors impact resilience. It would also be important to examine the influence of contextual and sociocultural factors on resilience. Ungar (2010) argues that resilience is nested in culture. Therefore, exploring medical educator resilience across cross-cultural contexts through both quantitative and qualitative research would be valuable to help fully understand this dynamic.

Finally, it is essential to explore how medical educator resilience might influence learners. This study has highlighted that medical educators have comparable levels of resilience to other populations and they identify multidimensional factors as influencing their resilience. How this translates into the formal, informal, and hidden curricula is worthy of further exploration.

## Conclusion

This study adds to the sparse research relating to medical educators globally. In particular, it examines resilience in a cohort of UK medical educators which has been previously unexplored. Participants’ resilience levels were found to be comparable to other population samples. A negative association between depressive symptoms and resilience levels was identified, although further research is needed to clarify this relationship.

Educators reported multidimensional factors that influenced their resilience. However, they predominately viewed internal factors as being supportive, and external factors as undermining. This study highlights the importance of considering contextual and sociocultural influences, alongside individual aspects when addressing resilience in the medical education community. Finally, it raises questions for further exploration about how medical educator resilience might influence the resilience of learners.

## Take Home Messages

• Medical educators’ resilience levels were found to be comparable to other population samples, and only depressive symptoms were found to be negatively associated with resilience levels.
• Educators perceived multidimensional influences on their own resilience, but predominately viewed internal factors as being supportive, and external factors as undermining.
• This study highlights the importance of considering external factors, such as contextual and sociocultural influences, in addition to individual factors when addressing resilience.
• Further research is needed to explore the external factors impacting resilience, and how medical educator resilience may influence learners through the formal, informal, and hidden curricula.

## Notes On Contributors

**Dr. Linda Chan**, is a family physician who recently completed a Master’s in Medical Education through the University of Dundee. Her research interests lie in developing effective educational strategies to foster resilience and well-being in learners. ORCID: https://orcid.org/0000-0002-9802-5059

**Dr. Ashley Dennis**, is the director for the Office of Medical Education, Billings Clinic. Working previously as a lecturer at the Centre for Medical Education at the University of Dundee, her research and pedagogical interests centre on how educational structures support health professionals’ development of resiliency and coping skills as they progress through their training.
