# Supplementary material for: Resilience: a nationwide study of medical educators
Source: MedEdPublish (2016). 2019 Aug 9;8:20. Originally published 2019 Jan 29. [Version 2] doi: 10.15694/mep.2019.000020.2 (PMC10712508; doi:10.15694/mep.2019.000020.2)
Supplement: Peer review history

# Reviewer report: Reviewer response for version 2

**DOI:** 10.21956/mep.20241.r31383

**Published:** 2019-08-11

**Authors:** Judy McKimm

**Affiliations:** 1 Swansea University

This review has been migrated. The reviewer awarded 4 stars out of 5 I did not read the earlier version of the paper, but it is a well written and constructed paper with some interesting messages about what shapes the resilience of medical educators. I think it is really interesting that (in general) internal factors support whereas external factors undermine individual resilience and this definitely needs further exploration in various contexts as it is somewhat differs from other studies, it maybe that the sample was biased (as the authors mention) but this is interesting to explore as would be the influence of educator resilience on students because we know the influence of leaders on their teams in strengthening or lessening team resilience and performance.

Reviewer Expertise:

NA

No decision status is available

# Reviewer report: Reviewer response for version 2

**DOI:** 10.21956/mep.20241.r31381

**Published:** 2019-08-11

**Authors:** Ken Masters

**Affiliations:** 1 Sultan Qaboos University

This review has been migrated. The reviewer awarded 5 stars out of 5 A well-constructed revision of an already very good paper. The authors have done well to incorporate the reviewers’ comments and maintain the overall flow of the paper. Well done.

Reviewer Expertise:

NA

No decision status is available

# Reviewer report: Reviewer response for version 2

**DOI:** 10.21956/mep.20241.r31382

**Published:** 2019-08-10

**Authors:** Balakrishnan Nair

**Affiliations:** 1 Centre for Medical Professional Development and University of Newcastle

This review has been migrated. The reviewer awarded 5 stars out of 5 This is a very good paper on a very important topic . Medical educators are unsung heroes. They teach, take care of patients, do research and have families . In my experience , a minority of people do the majority of teaching in most institutions. In our system in Australia, most of the clinical teaching is done by clinicians , employed by the health system with honorary appointments with the medical school. I often wondered how they cope with the work load . So I found this study very interesting . I would like to know whether these teachers , were employed to teach or were honorary teachers ? How many hours were spent on teaching and how many hours on other work related activities ? It would have been interesting to find out the time spent on exercise, meditation or mindfulness and other “stress relieving items” ( including alcohol consumption )I am sure these will be included in their next project

Reviewer Expertise:

NA

No decision status is available

# Reviewer report: Reviewer response for version 2

**DOI:** 10.21956/mep.20241.r31380

**Published:** 2019-08-10

**Authors:** Trevor Gibbs

**Affiliations:** 1 AMEE

This review has been migrated. The reviewer awarded 5 stars out of 5 As before, I thought that this was a very valuable paper to add to the literature on the subject and I would continue to recommend it to all involved in curricula and student development. The authors have addressed the minor concerns expressed by the reviewers, although I am not sure that they were really major issues, and by doing so have improved the paper.I now look forward to the next piece of research that comes from this group of authors

Reviewer Expertise:

NA

No decision status is available

# Reviewer report: Reviewer response for version 2

**DOI:** 10.21956/mep.20241.r31384

**Published:** 2019-08-09

**Authors:** Felix Silwimba

**Affiliations:** 1 University of Lusaka

This review has been migrated. The reviewer awarded 5 stars out of 5 such studies that interrogate the physical and mental health of medical educators are good. this is because the health of the educators is important for learners

Reviewer Expertise:

NA

No decision status is available

# Reviewer report: Reviewer response for version 1

**DOI:** 10.21956/mep.19121.r27596

**Published:** 2019-02-05

**Authors:** Johnny Lyon Maris

**Affiliations:** 1 Southampton GP Education Unit

This review has been migrated. The reviewer awarded 3 stars out of 5 This is a topical subject and the paper adds to the knowledge available. I am interested in the participants; were they undergrad or post grad educators (important as their line management structures in the UK work in very different ways and also in post grad medical education is usually part of a portfolio - the education adding resilience to their clinical work, whereas undergrad it is sometimes the other way round). I think the distress needs to be interrogated more, is it their primary clinical work that is causing distress or their medical educator role? Was there a difference between medically qualified and non-medically qualified educators or part-time vs whole time medical educators. It leaves many questions open and analysis of the roles of the educators (if available) will add more information.

Reviewer Expertise:

NA

No decision status is available

# Reviewer report: Reviewer response for version 1

**DOI:** 10.21956/mep.19121.r27599

**Published:** 2019-02-03

**Authors:** Nandalal Gunaratne

**Affiliations:** 1 Faculty of Medicine, Wayamba University of Sri Lanka

This review has been migrated. The reviewer awarded 4 stars out of 5 Well written paper and ncontemporary topic. However the findings seems to show that resilience is not an issue as a cause for the introductory statement "Among medical learners and practitioners, alarming rates of psychological distress have garnered attention in recent years"Therefore we need to find what is.

Reviewer Expertise:

NA

No decision status is available

# Reviewer report: Reviewer response for version 1

**DOI:** 10.21956/mep.19121.r27595

**Published:** 2019-02-02

**Authors:** sathyanarayanan varadarajan

**Affiliations:** 1 SRM Medical College, SRM University

This review has been migrated. The reviewer awarded 4 stars out of 5 This fascinating article/study explores resilience and the factors that relate to resilience in UK medical educators..The study was conducted by employing a cross-sectional online questionnaire, collecting sociodemographic characteristics and gathering educators’ views through open-ended questions in 247 educators. Data was analysed using quantitative (e.g. multiple regression analysis), and qualitative (i.e. thematic framework analysis) approaches.The authors found out that increasing depressive symptoms were associated with decreasing resilience levels in medical educators and they identified multidimensional factors that influenced their resilience. Another interesting finding is that respondents mainly considered internal resources as being resilience promoting, while external factors were undermining.This study highlights the importance of considering external factors, such as contextual and socio-cultural influences, in addition to individual factors when addressing resilience. The authors also aptly describe the future directions for research in this area.Though there are several limitations in this study, still it significantly adds to the sparse research relating to medical educators globally.

Reviewer Expertise:

NA

No decision status is available

# Reviewer report: Reviewer response for version 1

**DOI:** 10.21956/mep.19121.r27598

**Published:** 2019-01-30

**Authors:** Pamela Houghton DeVoe

**Affiliations:** 1 University of New Mexico School of Medicine

This review has been migrated. The reviewer awarded 4 stars out of 5 This paper presents an interesting angle to an increasingly salient topic in medical education. Medical student and educator well-being continues to garner attention in graduate health science training, as curricular density and complexity challenge participants’ ability to balance responsibilities. This well written paper adds depth to the discussion using a mixed methods approach, with two validated self-report surveys, and an open response component. The problem description is well referenced, Of interest is the participants reporting of internal factors being most likely to build resilience and external factors most likely to diminish resilience. Results, and hopefully additional research, suggest avenues for institutions to take a more purposeful approach to building environments that encourage resilience in students and educators.

Reviewer Expertise:

NA

No decision status is available

# Reviewer report: Reviewer response for version 1

**DOI:** 10.21956/mep.19121.r27594

**Published:** 2019-01-29

**Authors:** Ken Masters

**Affiliations:** 1 Sultan Qaboos University

This review has been migrated. The reviewer awarded 4 stars out of 5 A well written paper. The Introduction positions the paper well within the literature on resilience, both generally and in the medical education context. The Methods of data gathering and analysis are very well described. The Discussion reflects on the literature and details the contribution of this research to our knowledge. One weakness is in the reporting on the qualitative data. I would really like to have seen many more examples of comments from the participants. The space in the journal allows for it, and the authors should have utilised it. If the authors elect to create a version 2 of this paper (and I hope they do), I would like to see more examples. If they are concerned about interrupting the flow of the paper, then they could supply these examples as part of the Appendix.Two minor issues:• As far as I know, the full name of the PHQ-8 is “Patient Health Questionnaire Depression Scale”, and so, it might be best to use that full name when first mentioning (rather than simply “Patient Health Questionnaire”).• “Data was” should be “Data were”.

Reviewer Expertise:

NA

No decision status is available

# Reviewer report: Reviewer response for version 1

**DOI:** 10.21956/mep.19121.r27593

**Published:** 2019-01-29

**Authors:** Judy McKimm

**Affiliations:** 1 Swansea University

This review has been migrated. The reviewer awarded 4 stars out of 5 This is an interesting study on a very topical area of medical education. I think that it is a bit of a different slant to use mixed methods and 2 survey tools as this adds to the richness of the data. I would have liked some more deeper analysis around some of the findings which lead onto further exploration. There are some useful models in the literature around the factors that contribute to personal resilience, which could be explored further, ideally in different cultural contexts, as this might vary. Picking up Kathleen's point, consideration of organisational, cultural and team resilience is under researched in our field and that would be fascinating to explore.

Reviewer Expertise:

NA

No decision status is available

# Reviewer report: Reviewer response for version 1

**DOI:** 10.21956/mep.19121.r27592

**Published:** 2019-01-29

**Authors:** Kathleen Leedham-Green

**Affiliations:** 1 King's College London / Imperial College London

This review has been migrated. The reviewer awarded 5 stars out of 5 This is a well conducted review of resilience amongst UK medical educators. It has some important quantitative results, both positive and negative: resilience not related to personal characteristics, and burnout is related to depression, though I feel there may a chicken and egg argument with the latter.Their qualitative findings were helpful and resonated with my knowledge of our clinical teaching network. Their conclusions are important and actionable: we need to look at ways to support a person's sociocultural and work context (external factors), rather than teach the person to be stronger (internal factors).I would love to see the authors expand this research to generate a survey based on their qualitative findings - getting educators to rate factors according to how they perceive their impact on their personal resilience. Could they run an ANOVA between depressed and non-depressed educators (and resilient/non-resilient educators) to see which external factors are most predictive of burnout?

Reviewer Expertise:

NA

No decision status is available

# Reviewer report: Reviewer response for version 1

**DOI:** 10.21956/mep.19121.r27597

**Published:** 2019-01-29

**Authors:** Trevor Gibbs

**Affiliations:** 1 AMEE

This review has been migrated. The reviewer awarded 4 stars out of 5 An interesting piece of work and very valuable for the future. Resilience is an important topic, placed withing our changing world of healthcare and healthcare education. I was particularly interested in whether the life of medical educators was the same as those with a pure clinical role, my impression being that having two such roles was more demanding that one. I am not sure that I could deduce this though from the paper, and some of the comments in the paper related more to the clinical aspect of the job rather than the medical educator ones.An important paper for all those involved in medical education

Reviewer Expertise:

NA

No decision status is available
